# PRKDC regulates cGAMP to enhance immune response in lung cancer treatment

**DOI:** 10.3389/fimmu.2024.1497570

**Published:** 2024-11-26

**Authors:** Zhanghao Huang, Runqi Huang, Jun Zhu, Youlang Zhou, Jiahai Shi

**Affiliations:** ^1^ Medical School of Nantong University, Nantong University, Nantong, China; ^2^ Department of Thoracic Surgery, Affiliated Hospital of Nantong University, Nantong, China; ^3^ Nantong Key Laboratory of Translational Medicine in Cardiothoracic Diseases, and Research Institution of Translational Medicine in Cardiothoracic Diseases, Affiliated Hospital of Nantong University, Nantong, China; ^4^ Research Center of Clinical Medicine, Affiliated Hospital of Nantong University, Nantong, China

**Keywords:** lung cancer, CGAMP, PRKDC, immunotherapy, STING pathway

## Abstract

**Background:**

Despite its involvement in nucleotide metabolism, tumor immune landscape, and immunotherapy response, the role of 2’-3’-cyclic guanosine monophosphate–adenosine monophosphate (2’,3’-cGAMP) in lung adenocarcinoma (LUAD) remails unelucidated. This study aimed to investigate the antitumor effects of 2’,3’-cGAMP in LUAD.

**Method:**

Herein, patients with LUAD were screened for prognostic biomarkers, which were then assessed for sensitivity to immunotherapy and chemotherapy utilizing the “TIDE” algorithm and CellMiner database. The results were validated using a mouse xenograft model. Additionally, macrophages and lung cancer cells were co-cultured, and macrophage polarization and apoptosis levels in the lung cancer cells were detected through flow cytometry. Protein levels were analyzed through western blotting and immunofluorescence. Finally, drug-encapsulated nanoparticles were designed to systematically examine the antitumor efficacy of the treatment against LUAD.

**Result:**

Notably, 2’,3’-cGAMP-mediated protein kinase, DNA-activated, catalytic subunit (PRKDC) inhibition induced macrophage polarization toward the M1 phenotype, thereby triggering apoptosis in LUAD cells. Furthermore, *in vivo* experiments showed that M1 macrophage infiltration enhancement and apoptosis induction in lung cancer cells were achieved by suppressing PRKDC expression via 2’,3’-cGAMP, which inhibited lung cancer growth. The machine-learning approaches revealed SB505124 to be an effective antitumor agent in LUAD cells with high PRKDC levels owing to its ability to promote 2’,3’-cGAMP-mediated apoptosis. Encapsulation of 2’,3’-cGAMP, and SB505124 within a nano-delivery system markedly reduced tumor volumes in murine lung cancer tissues compared with that by individual agents.

**Conclusion:**

The findings of this study reveal that PRKDC can predict poor survival of patients with LUAD. Additionally, SB505124 enhances the efficacy of 2’,3’-cGAMP-based immunotherapy in patients exhibiting a high PRKDC expression.

## 1 Introduction

Lung cancer is the leading cause of cancer-related mortality worldwide, and LUAD is among its most prevalent subtypes. Although various advances have been made in chemotherapeutic and targeted treatment modalities, the prognosis for patients with LUAD remains poor, underscoring an urgent need for novel therapeutic strategies and reliable prognostic biomarkers (1). The development of therapies targeting molecular aberrations within tumors offers a promising avenue for improving treatment outcomes, necessitating the identification and elucidation of key molecular drivers of tumor progression and treatment response (2).

Nucleotide metabolism has been intricately associated with the rapid proliferation of tumor cells, facilitating DNA and RNA synthesis (3). Consequently, disrupting such metabolic pathways can lead to the accumulation of oncogenic mutations and disrupt cellular energy status, affecting cancer progression (4). Reportedly, alterations in nucleotide metabolism pathways can promote rapid cell division and cell death resistance in lung cancer (5). The cyclic dinucleotide 2’-3’-cyclic guanosine monophosphate (GMP)–adenosine monophosphate (AMP) (2’,3’-cGAMP), a product of nucleotide metabolism, has been reported as a critical mediator (6, 7), and it affects various cellular processes by acting as a secondary messenger in immune signaling pathways, thereby modulating the tumor microenvironment (TME) and potentially inhibiting tumor progression (8, 9).

Reportedly, 2’,3’-cGAMP plays a significant immune-modulatory role, especially regarding immune cells and inflammatory cytokines (10). It is a potent inducer of the stimulator of interferon genes (STING) pathway, and enhances the innate immune response, particularly by activating the M1 phenotype polarization of macrophages, which is associated with antitumor activity (11, 12). The STING pathway triggers inflammation and affects the recruitment and function of various immune cells within the TME (13). Nevertheless, the underlying specific mechanisms by which 2’,3’-cGAMP regulates immune responses in lung cancer remain poorly understood, necessitating further studies to explore its therapeutic potential.

This study aimed to investigate the therapeutic potential of 2’,3’-cGAMP in LUAD. The experiments focused on the interaction of 2’,3’-cGAMP with the immune system and its potential to modulate immune-mediated tumor suppression. Furthermore, multi-omics analyses and functional assays were employed to investigate the role of protein kinase, PRKDC, a key enzyme involved in DNA repair, in modulating the effects of 2’,3’-cGAMP. Lastly, the study explored the sensitizing effects of SB505124, a drug identified through machine-learning approaches, to potentiate the antitumor activity of 2’,3’-cGAMP, thereby providing a research basis for developing novel combinatorial approaches for treating LUAD.

## 2 Materials and methods

### 2.1 Cell lines and mice

The experiment protocols employed in this study were approved by the Experimental Animal Care and Use Committee of Nantong University (approval number: IACUC20230616-1003). The inclusion of LUAD tissues in experiments was approved by enrolled patients and the Research Ethics Committee of Nantong University Affiliated Hospital (approval number: 2020-L002).

Lung cancer cell lines, including LLC, were obtained from the Chinese Academy of Sciences. Additional lung cancer cell lines, such as LA795 and H1299, and macrophage cell lines RAW 246.7 and THP-1 were procured from the Cell Center of Peking Union Medical College, Beijing, China (detailed information available at http://cellresource.cn/cellsearch.aspx). ICR mice were acquired from the Nantong University Animal Center (approval number: IACUC20230616-1003).

### 2.2 Cell apoptosis detection

Lung cancer cells were co-cultured with macrophages and then resuspended in the binding buffer. After a 10-min incubation at 37°C under 5% CO_2_, propidium iodide (PI) and Annexin-V antibody staining were performed under dark conditions for 15 min. The Attune NxT system (Invitrogen), operated with the Attune NxT software version 2.7.0, was used for analyzing all specimens through the PI-A and Annexin V-fluorescein isothiocyanate channels.

### 2.3 Drug treatment in mice

Herein, subcutaneous tumors were developed on the right side of mice by implanting 100,000 LLC-luc mouse lung cancer cells. At day 5 post-tumor implantation, treatments with 2’,3’-cGAMP (catalog HY-100564A, MCE, USA) and SB505124 (catalog HY-13521, MCE, USA) were commenced. Mice were locally administered with 0.1 mg/kg 2’,3’-cGAMP combined with intraperitoneal administration of 0.5 and 1 mg/kg drug for 2 weeks. Following this, *in vivo* imaging was performed using the IVIS Spectrum system (PerkinElmer Health Sciences, USA) (14).

### 2.4 Macrophage polarization detection

Approximately 10,000 macrophages (macrophage cell lines: THP-1 and RAW 246.7) were seeded per 190,000 lung cancer cells in each well of 24-well plates and treated with various doses of SB505124 and 2’,3’-cGAMP. Following the application of cluster of differentiation 206 and fluorescent secondary antibodies, macrophage polarization was evaluated by flow cytometry (Attune NxT, Invitrogen) after a 24-h incubation (15).

### 2.5 Western blotting

The phenylmethylsulfonyl fluoride-containing radioimmunoprecipitation assay lysate (100:1) was cooled and then mixed with the loading buffer. Next, constant-volume protein samples were electrophoresed and then electroblotted onto a polyvinylidene fluoride membrane using a 400-mA current. After sealing the membrane for 20 minutes using a very effective sealer, the membrane was incubated with various primary antibodies, including anti-β-actin and anti-B-cell lymphoma-2-associated X protein (Bax), overnight at 4°C. Following incubation, the membrane was washed with Tris-buffered saline with Tween 20 thrice (10 min per wash). Following an overnight incubation at 4°C, IRDYE800-conjugated secondary antibody was added. The results were visualized using the Odyssey infrared imaging system (LiCOR, Lincoln, NE), and a computerized imaging system was used for the quantitative analysis of band intensities.

### 2.6 Immunohistochemistry assay

Tumor specimens preserved in a 10% formalin solution at room temperature for 3 d were embedded in paraffin. Subsequently, 5-μm-thick sections were meticulously cut, affixed to positively charged slides, and heated at 60°C for 10 min to facilitate degreasing and rehydration. The slides were then treated with a specific retrieval buffer and heated to 110°C for 17 min before being allowed to cool to ambient temperature to retrieve the antigens. After washing the tissues thrice with phosphate-buffered saline (PBS), each wash of 5 min, the sections were sealed for 30 min, followed by another 5-min PBS rinsing. Next, the samples were incubated with primary antibodies overnight at 4°C, washed thrice with PBS for 5 min each, and incubated with secondary antibodies at room temperature for 2 h. Finally, 3,3’-diaminobenzidine staining of tissue samples was performed, and target proteins were visualized using an inverted confocal fluorescence microscope (Fluoview FV1000, Olympus). The quantitative analyses were performed utilizing the Image J software (16).

### 2.7 Immunofluorescence analysis

Herein, tissues derived from mouse lung cancer and their co-cultured cells were fixed using 4% paraformaldehyde and then permeabilized. The samples were added with primary antibodies and Alexa-488- or Alexa-546-conjugated secondary antibodies (specific for mouse or rabbit immunoglobulins) for staining. Nuclei were stained using 4’,6-diamidino-2-phenylindole. The stained samples were imaged using an Olympus Fluoview FV1000 inverted confocal fluorescence microscope. The Image J software was used to analyze the images quantitatively.

### 2.8 Synthesis of drug-encapsulated nanoparticles

The double emulsion solvent evaporation technique was used to prepare the nanoparticles. First, 200 µL of dimethyl sulfoxide (DMSO) containing 10 mg of either 2’,3’-cGAMP and SB505124 or indocyanine green was mixed with 1 mL of dichloromethane. This mixture was added with 100 mg of poly(lactic-co-glycolic acid) (PLGA; lactic acid-to-glycolic acid ratio: 65:35, molecular weight: 40,000–75,000; Sigma-Aldrich). Next, the mixture was sonicated in a 3 mL solution of 7% polyvinyl alcohol (PVA) using an ultrasonic homogenizer (BE, Germany) for 30 seconds in an ice bath to obtain an initial emulsion (molecular weight: 14,160). This primary emulsion was then transferred into 1% PVA solution (50 mL) and sonicated for an additional 1 min to form a secondary emulsion, which was continuously stirred for at least 24 h at room temperature to allow complete evaporation of dichloromethane. The nanoparticles were isolated and purified by centrifuging the emulsion thrice at 15,000 rpm for 5 min, along with three successive rounds of washing with distilled water. The purified nanoparticles were finally resuspended in deionized water to be used for further analyses (17).

### 2.9 STING signature-based LUAD prognosis model

The distinct modification patterns of STING were identified utilizing an unsupervised clustering methodology. The robustness and counts of these clusters were verified through consensus clustering, which executed 1,000 iterations to guarantee stability in classification (18). The STING-associated hazard ratio for prognosis was calculated using a univariate Cox regression model. Moreover, Lasso–Cox techniques were employed to identify the independent prognostic factors. The risk scores for patients with LUAD were established based on a prognostic signature that included six STING pathway-related genes (19).

### 2.10 ImmuneScore and immunological correlation analyses

The ImmuneScore was evaluated using the R package ‘Gene Set Variation Analysis’ (GSVA), facilitating the identification of distinct cell types (20). Subsequently, the ‘TIDE’ algorithm was used to compute the immune scores for individual samples. Additionally, the antitumor drugs were screened using the ‘CellMiner’ tool (21). Summary statistics of exposed genetic instrumental variables and results of genome-wide association studies (GWAS) were from OpenGWAS, developed by the MRC IEU OpenGWAS Project, a contributor to TwoSampleMR. The criteria of SNP identification were as follows: P = 5 × 10^−6^, kb = 1,000, r^2^ = 0.01.

### 2.11 Statistical analyses

Comparative analyses of continuous variables between two cohorts were performed using Student’s t-tests. All statistical analyses were bidirectional, and the P-value of <0.05 was considered statistically significant. * denotes P < 0.05, ** denotes P < 0.01, and *** denotes P < 0.001.

## 3 Results

### 3.1 Development of LUAD prognostic model via STING pathway-related genes

**Figure 1:**
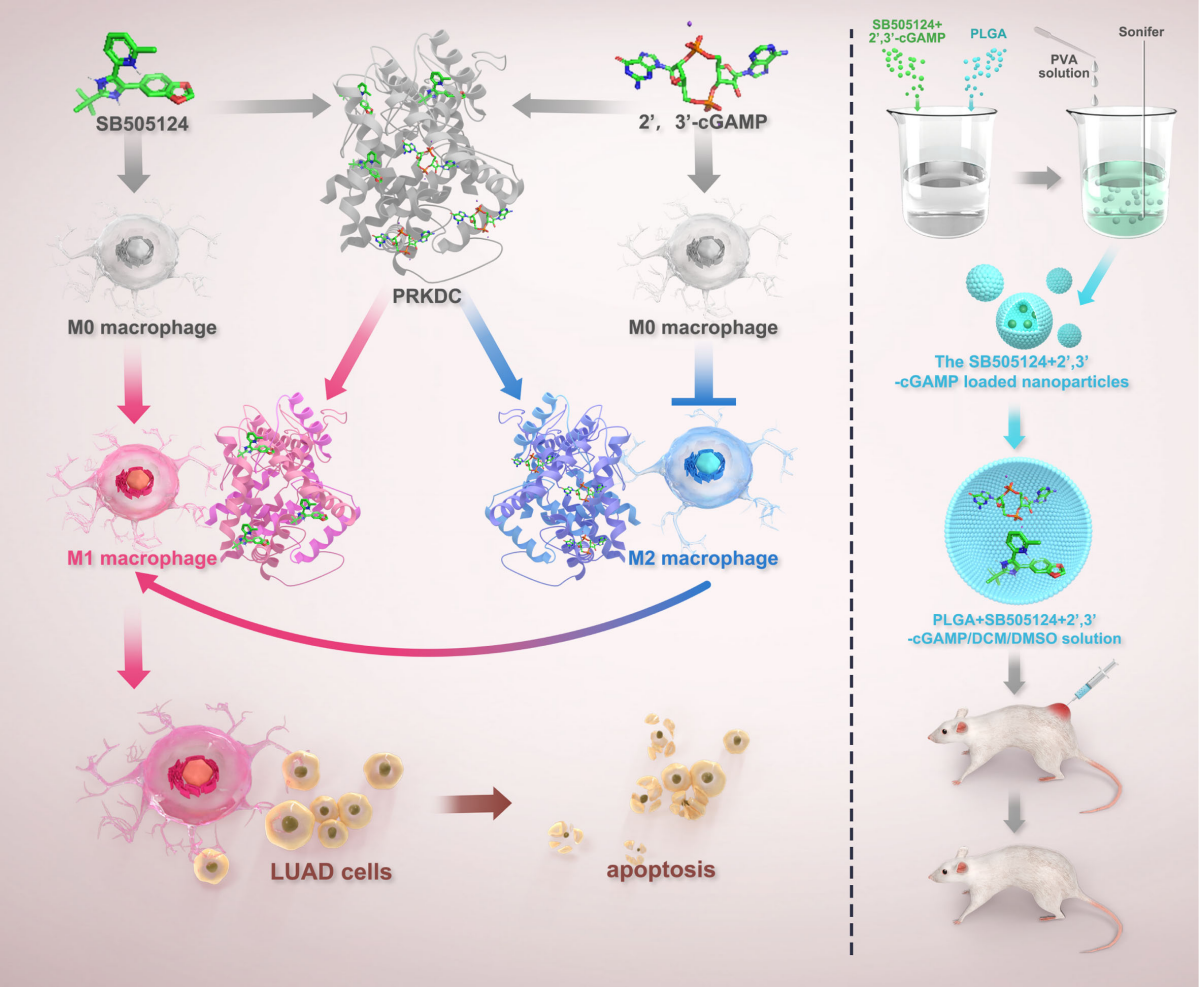
The schematic of the study design.

**Figure 2:**
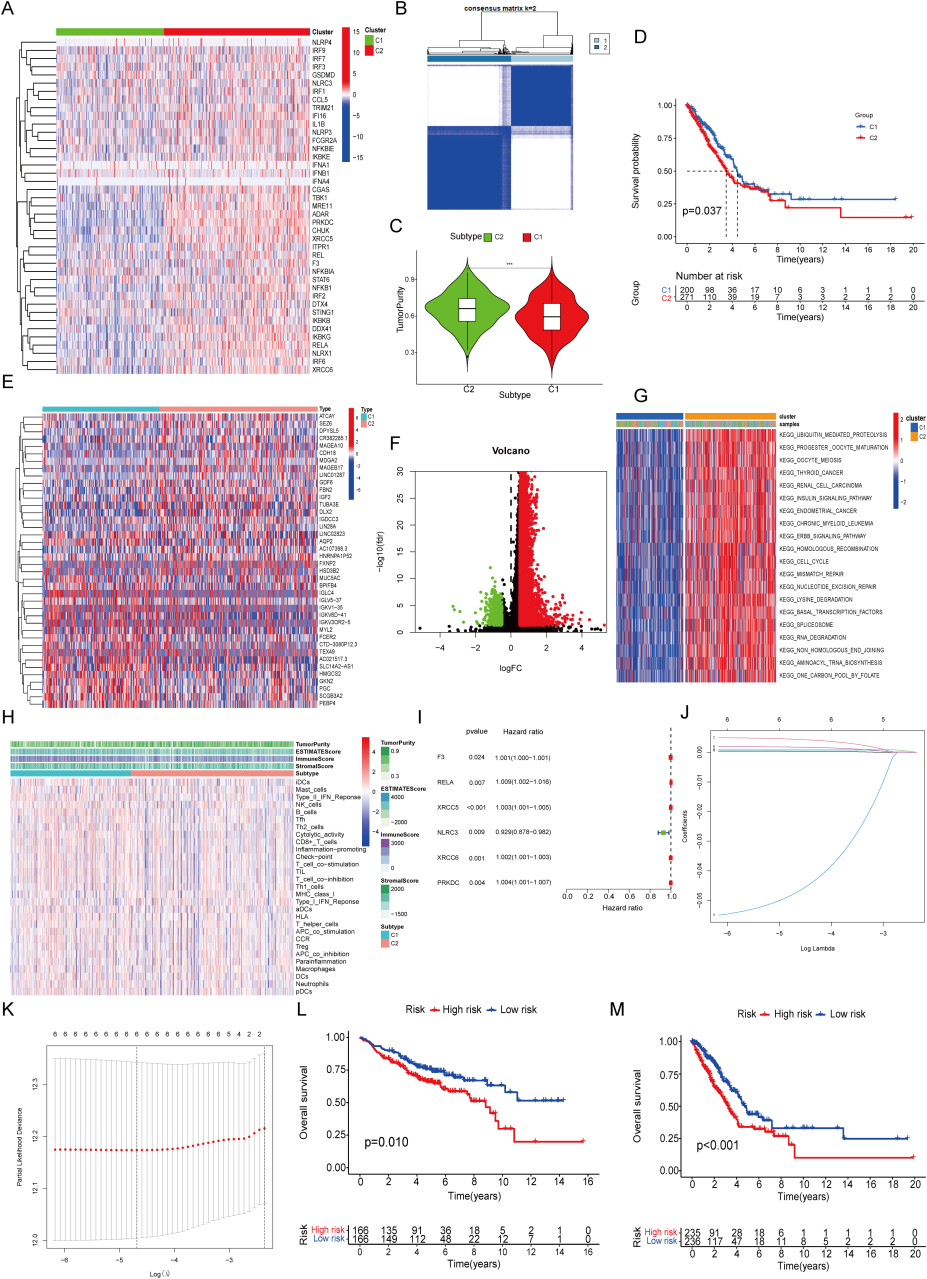
Unsupervised Cluster Analysis and Signature Construction. **(A)** Expression of 41 STING-related genes. **(B)** Unsupervised cluster analysis. **(C)** Tumor purity. **(D)** Survival curve. **(E)** DEGs between the two clusters. **(F)** Volcano map of DEGs. **(G)** Functional enrichment of GSVA. **(H)** The immune characteristics analyzed by ssGSEA. **(I)** Single factor regression analysis. **(J, K)** Lasso regression analysis. **(L)** Survival curve of GSE37745 combined with GSE31210. **(M)** Survival curve of TCGA.

The overall study design is schematically presented in **Figure 1**. From the Kyoto Encyclopedia of Genes and Genomes database, 41 STING pathway-associated genes were identified and their expression profiles were subsequently examined within The Cancer Genome Atlas (TCGA) dataset (**Figure 2A**). Utilizing these genes, patients with LUAD were divided into two primary clusters. Based on consistency matrices generated by the consensus clustering algorithm, which performed 1,000 iterations to ensure classification stability, two optimal subtypes were determined (**Figure 2B**). Compared with the first cluster, the second subgroup exhibited increased tumor purity (**Figure 2C**). The results of survival analysis revealed that patients in the first subgroup exhibited markedly better outcomes than those in the second subgroup, which correlated with the lower tumor purity observed in the first subgroup (**Figure 2D**). Differentially expressed genes (DEGs) between the two clusters were visualized using volcano and heat maps (**Figures 2E, F**), and GSVA revealed their primary involvement in pathways related to homologous recombination and the cell cycle (**Figure 2G**). Furthermore, the second subgroup presented higher scores for tumor purity and stromal content, whereas the first subgroup exhibited enhanced immune responses. The abundance of macrophages was higher in the first subgroup (**Figure 2H**). Univariate Cox regression analysis identified six STING pathway-related genes with notable prognostic values (**Figure 2I**). To avoid overfitting in the biomarker-determining process, Lasso regression was employed, which screened six critical genes (**Figures 2J, K**), which were subsequently validated through multivariate Cox regression, namely coagulation factor III, v-rel reticuloendotheliosis viral oncogene homolog A, X-ray repair cross-complementing (XRCC)5, NOD-like receptor family CARD domain containing 3, XRCC6, and PRKDC as the top six prognostic markers. The GSE37745 and GSE31210 datasets were used for validation (**Figure 2L**) and TCGA dataset for training (**Figure 2M**). Patients were categorized into high- and low-risk (236 and 235 patients, respectively) groups based on median risk scores.

### 3.2 Validation of risk stratification in independent cohorts

**Figure 3:**
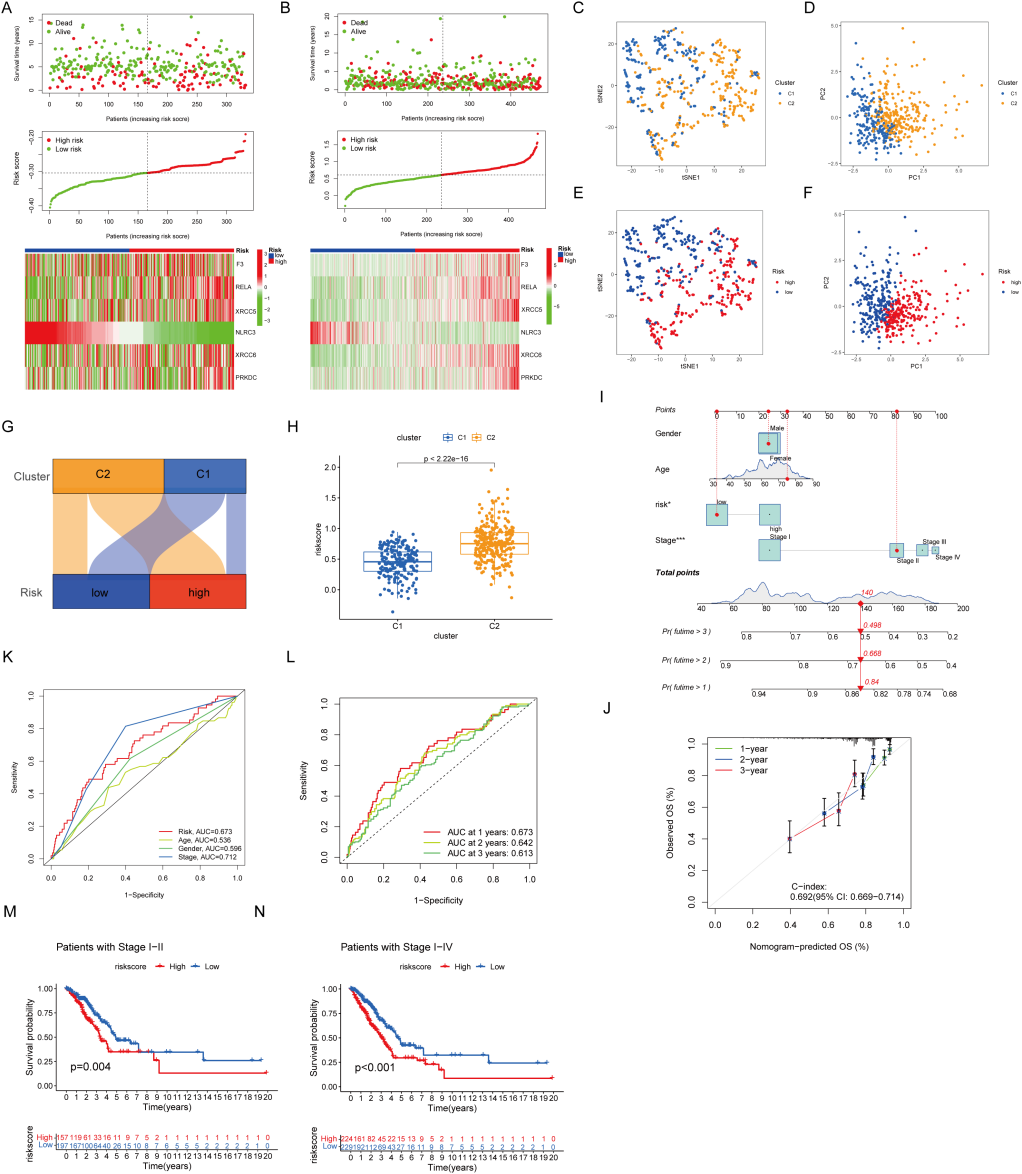
Nomogram prognostic signature. **(A)** Prognostic signature for GSE37745 and GSE31210. **(B)** Present prognostic signature for TCGA. **(C, E)** tSNE dimension reduction distribution. **(D, F)** PCA dimension reduction distribution. **(G)** Sankey plot. **(H)** The box diagram showed that cluster 2 has a higher risk score. **(I)** Diagrams properties of prognostic models and clinical features. **(J)** Reliability of the prognostic signature. **(K, L)** ROC curves of prognostic models. **(M, N)** Associations between prognostic models and clinical factors.

The validation cohort replicated the initial classification, comprising 166 patients assigned to each risk category based on median risk scores (**Figures 3A, B**) (22, 23). Dimensionality reduction methods, including t-distributed Stochastic Neighbor Embedding and principal component analysis, mapped the distribution and prognostic features of the clusters (**Figures 3C–F**). The relationship between identified clusters and risk groups was visualized through Sankey diagrams, revealing a strong association between Cluster 1 and the low-risk category and between Cluster 2 and the high-risk category (**Figure 3G**). The Cluster 2-associated increased risk score was further quantified (**Figure 3H**). The predictive performance of the prognostic signature was shown through a Norman diagram, which exhibited a concordance index (C-index) of 0.692, thereby affirming its reliability (**Figures 3I, J**). The receiver operating characteristic curve analysis further validated the accuracy of the prognostic signature (**Figures 3K, L**). Finally, stratified analysis of clinical outcomes revealed the improved survival rates of patients in the low-risk group at all stages I–IV compared with those in the high-risk group (**Figures 3M, N**).

### 3.3 Evaluation of immunotherapy outcomes and immune correlations

**Figure 4:**
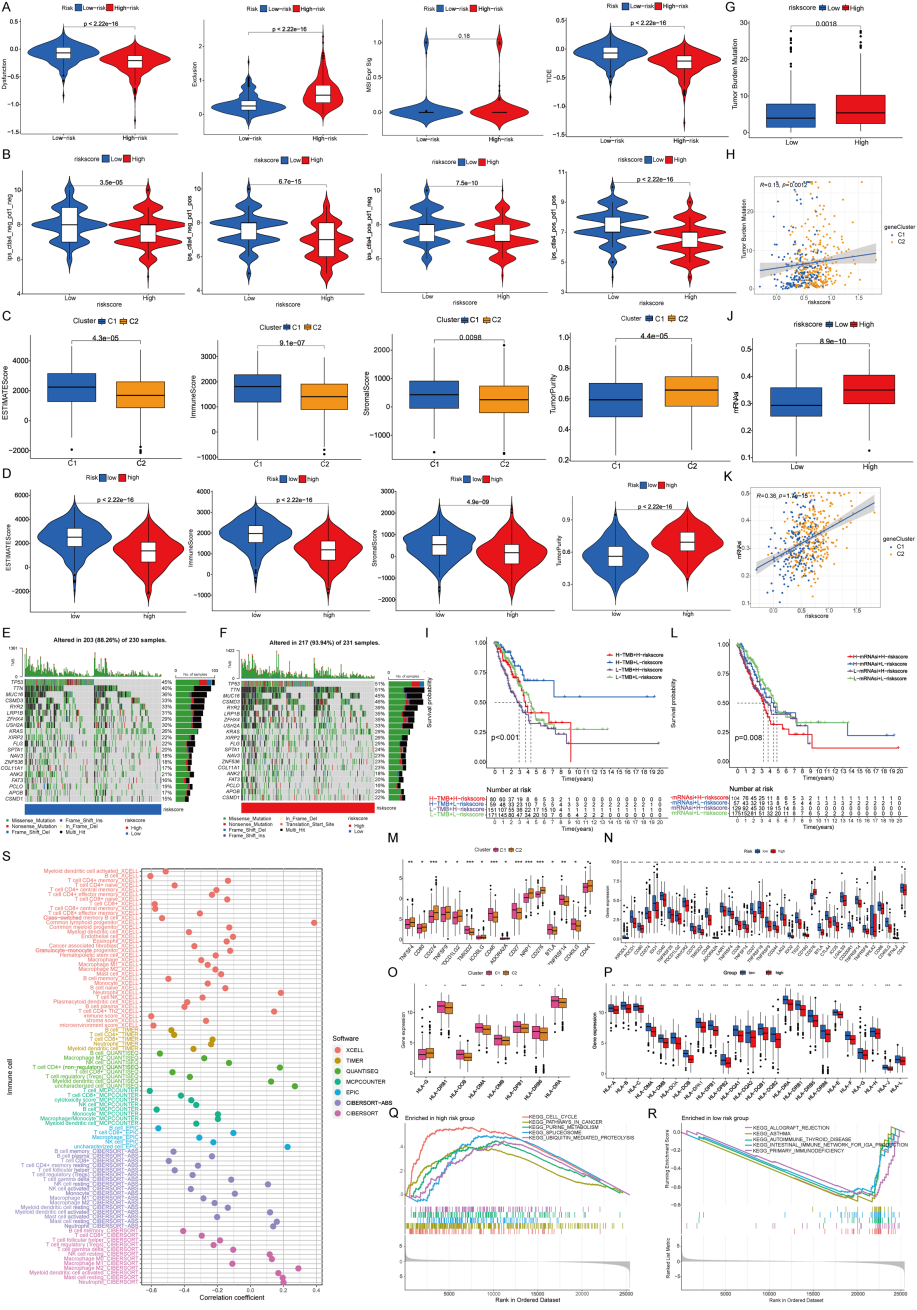
Immunological relevance of prognostic signature. **(A)** Immune escape capacity in prognostic signature. **(B)** Proportion of immune cell positives in prognostic signature. **(C)** Properties of immune microenvironment. **(D)** Characteristics of prognostic signature in immune microenvironments. **(E, F)** Tumor mutations. **(G-I)** TMB and prognostic models. **(J-L)** mRNAsi and prognostic models. **(M)** Differences of immune checkpoints between clusters. **(N)** Differences in immune checkpoints between prognostic signature. **(O)** Differences of co-stimulatory molecules between two clusters. **(P)** Differences in co-stimulatory molecules between prognostic signature. **(Q, R)** GSEA functional enrichment analysis of prognostic signature. **(S)** Immune infiltration in the prognostic signature among different immune algorithms.

To investigate the immunotherapy responses associated with identified prognostic signatures, the TIDE methodology was employed. Within the study cohort, the low-risk group exhibited increased dysfunction scores coupled with diminished exclusion rates (**Figure 4A**). The analysis of STING pathway-associated features revealed the immune surface phase score (IPS) across diverse therapeutic approaches. Violin plots illustrate the disparities in IPS between high- and low-risk groups within the training cohort (**Figure 4B**). Analysis of the immune microenvironment revealed that both the low-risk group and Cluster 1 possessed superior matrix and immune scores, whereas Cluster 2 and the high-risk group showed increased tumor purity (**Figures 4C, D**).

### 3.4 Somatic mutation and immune profile analysis

The results of somatic mutation analysis revealed an increased frequency of tumor protein 53 (TP53) and titin (TTN) mutations within the high-risk group (mutation rate: 51%), compared with the 45% prevalence of TP53 mutations in the low-risk group (**Figures 4E, F**). Immune profile analysis showed that the high-risk category, corresponding to Cluster 2, was associated with a higher tumor mutation burden (TMB). Notably, patients with high TMB in the low-risk group exhibited superior survival outcomes (**Figures 4G–I**). Additionally, the messenger RNA-based stemness index (mRNAsi) was found to be increased in both Cluster 2 and the high-risk group, whereas lower mRNAsi scores were associated with enhanced survival in the low-risk group (**Figures 4J–L**) (24).

Herein, differential expression analysis of immune checkpoint proteins revealed notable disparities between the risk groups (**Figures 4M, N**). Furthermore, human leukocyte antigen (HLA) exhibited differential expression in different groups (**Figures 4O, P**) (25). Gene set enrichment analysis highlighted a predominance of immune-related pathways in the low-risk group, whereas the high-risk group presented more activeness in tumor-centric pathways (26). Lastly, the immune infiltration assessment of the prognostic model showed consistent results across various immune algorithms, reinforcing the immune-related characteristics of the signature genes (**Figures 4Q–S**).

### 3.5 Relationship between PRKDC and 2’,3’-cGAMP

**Figure 5:**
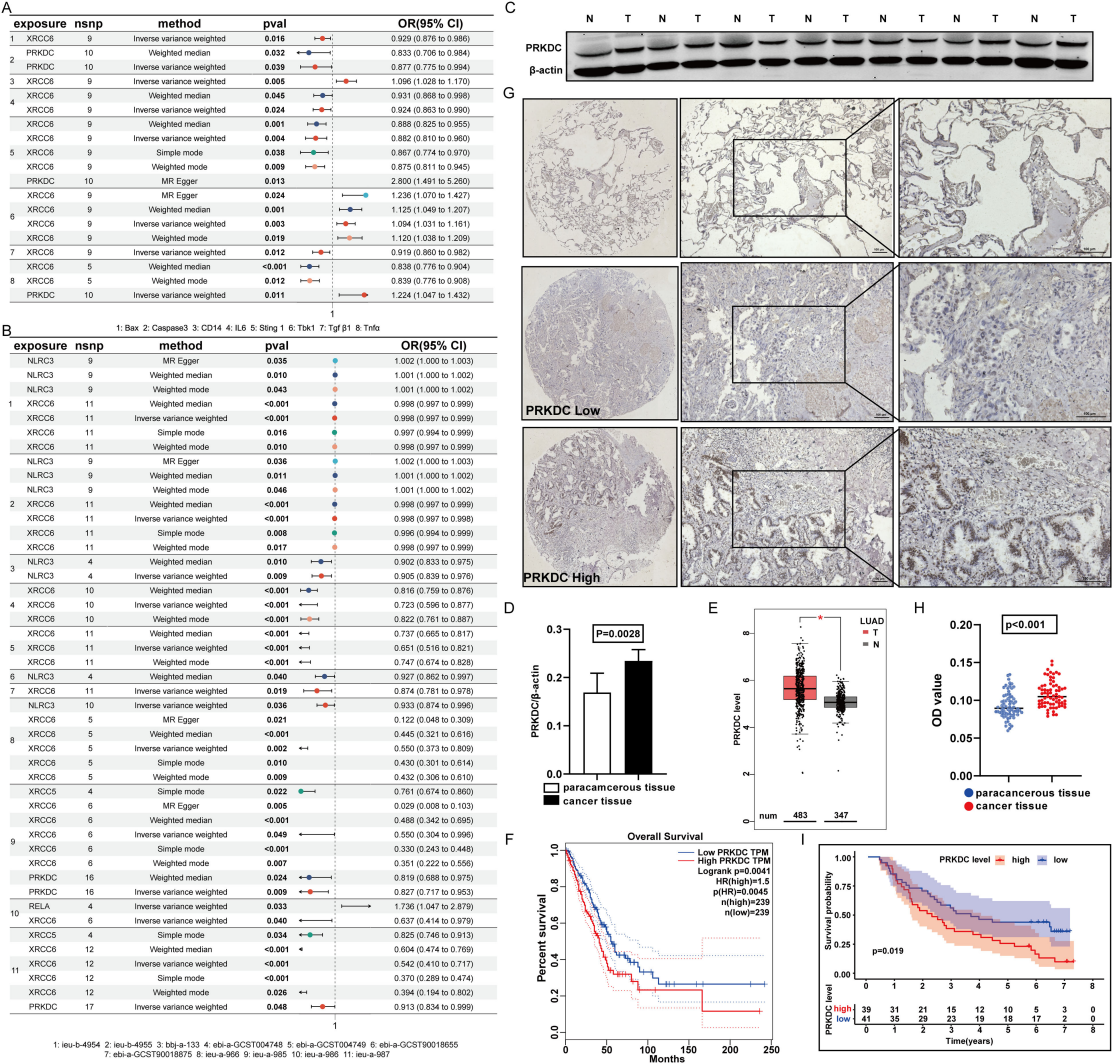
Features of PRKDC. **(A)** Screening of prognostic model genes and STING relationship. **(B)** Screen prognostic model genes for association with lung cancer. **(C, D)** The expression of PRKDC in cancer and adjacent tissues. **(E)** Screen prognostic model genes for association with lung cancer in TCGA. **(F)** Survival analysis of PRKDC in TCGA database. **(G-I)** Expression of PRKDC in 70 tissue pairs in immunomicroarray. **(I)** The survival of 70 pairs of patients according to PRKDC expression.

As instrumental variables, amino acid variations in the model genes XRCC6 and PRKDC considerably affected apoptotic protein levels, macrophage markers, and macrophage secretion factors (**Figure 5A**), thereby altering lung cancer outcomes (**Figure 5B**). Notably, compared with adjacent nontumor tissues, PRKDC expression in tumor tissues in LUAD was markedly upregulated (**Figures 5C, D**) (27). TCGA database analysis confirmed upregulated PRKDC expression in malignant tissues (**Figure 5E**), revealing its association with poorer prognostic outcomes in patients with lung cancer (**Figure 5F**). Consistently, validation within the independent LUAD cohort (n = 70) of this study further supported the findings that PRKDC expression in tumor tissues was upregulated (**Figures 5G, H**) and increased PRKDC levels correlated with poor patient survival (**Figure 5I**).

**Figure 6:**
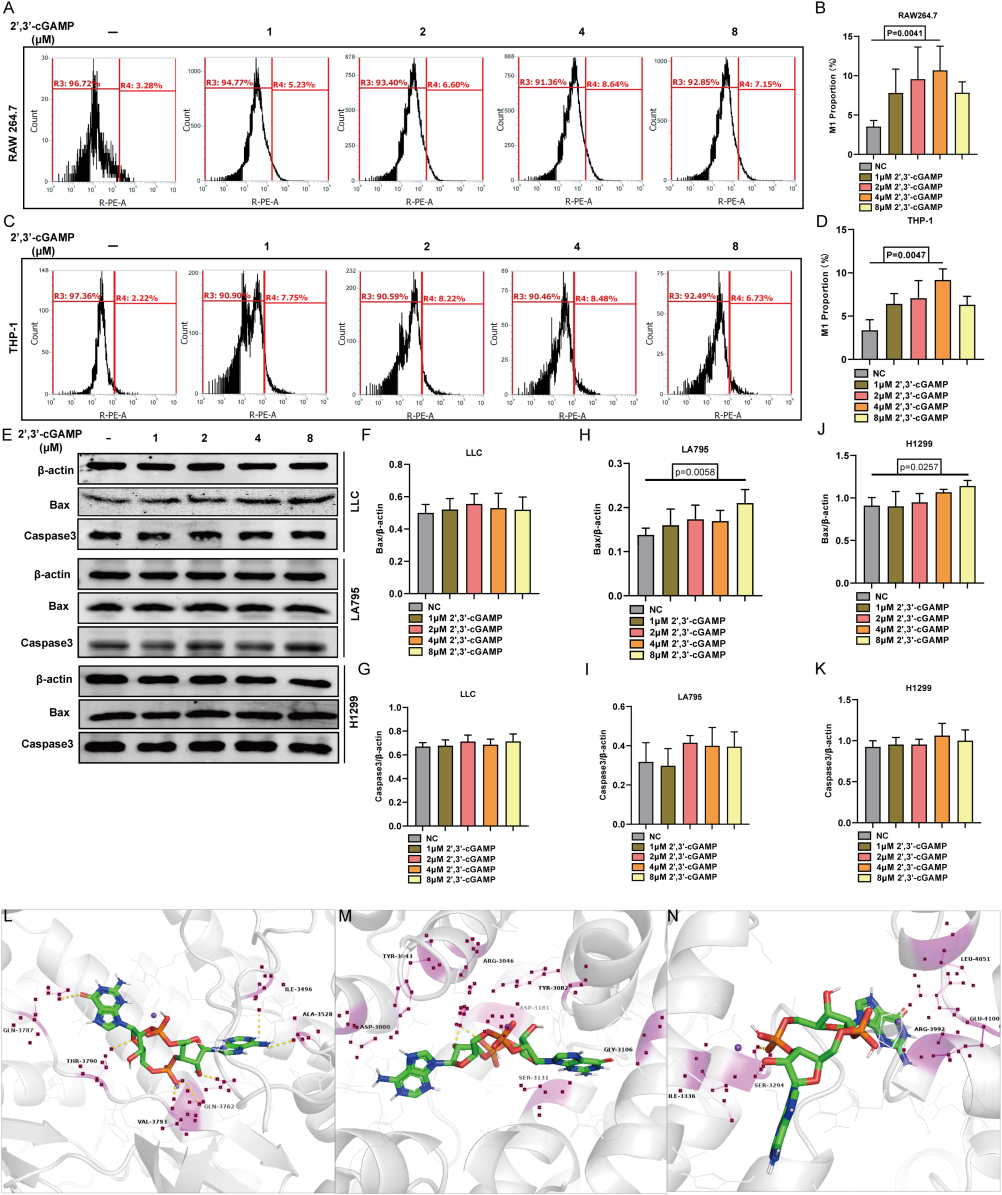
STING activator 2’,3’-cGAMP. **(A, B)** 2’,3’-cGAMP induced the polarization of RAW 264.7 into M1 macrophages. **(C, D)** 2’,3’-cGAMP induces THP-1 polarization into M1 macrophages. **(E-K)** 2’,3’-cGAMP induces apoptosis in lung cancer cells co-cultured with macrophages. **(L–N)** 2’,3’- Common position for cGAMP to interface with PRKDC.

The interaction between PRKDC and STING was elucidated by administering varying concentrations of the STING agonist 2’,3’-cGAMP to macrophage cell lines RAW264.7 and THP-1. Treatment with 4µM 2’,3’-cGAMP prompted the M1 polarization of macrophages (**Figures 6A–D**). The M1-polarized macrophages demonstrated cytotoxicity against multiple LUAD cell lines, including LLC, LA795, and H1299. With increasing 2’,3’-cGAMP concentration, apoptosis-related protein levels progressively increased (**Figures 6E–K**) (28). Potential binding sites were identified through molecular docking, revealing predominant amino acids, such as glutamic acid, valine, leucine, serine, tyrosine, and glycine, critical in facilitating the PRKDC–STING interaction (**Figures 6L–N**).

### 3.6 2’,3’-cGAMP inhibits LUAD cell growth *in vivo*

**Figure 7:**
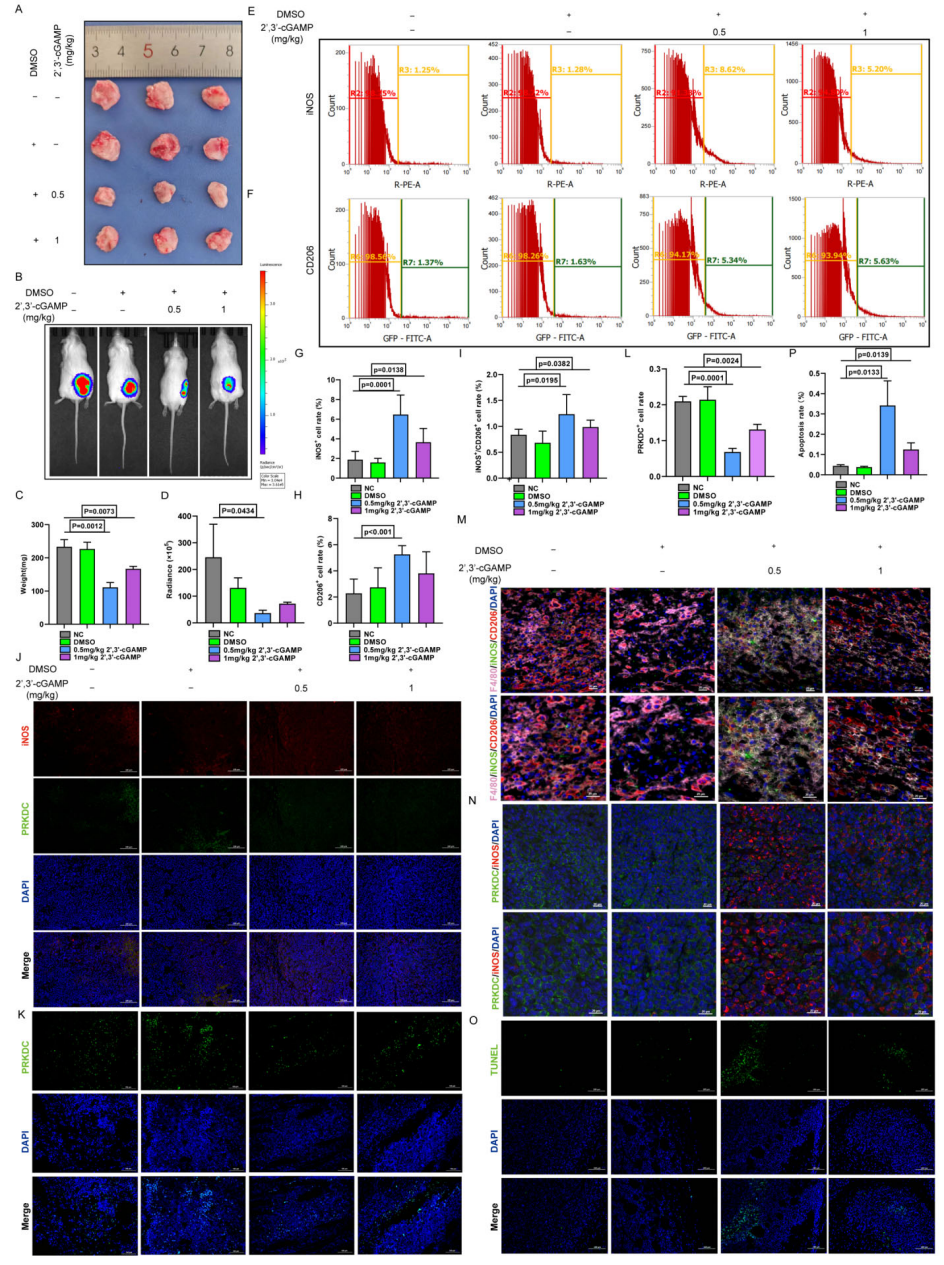
2’,3’-cGAMP promotes polarization of M1 macrophages. **(A)** Antitumor properties with 2’,3’-cGAMP. **(B)** Representative bioluminescence image of LLC-Luc. **(C)** Tumor mass changes after 2’,3’-cGAMP treatment. **(D)** Quantization of the bioluminescence image. **(E-I)** Macrophage polarization after 2’,3’-cGAMP treatment. **(J)** Immunofluorescence showed macrophages polarization and PRKDC changes after 2’,3’-cGAMP treatment. **(K, L)** Immunofluorescence analysis of PRKDC distribution after 2’,3’-cGAMP treatment. **(M)** Multicolor immunofluorescence. **(N)** Multicolor immunofluorescence showed the polarization of macrophages and the distribution of PRKDC after 2’,3’-cGAMP treatment. **(O, P)** Immunofluorescence of tumor apoptosis.

The effects of 2’,3’-cGAMP on LUAD were further elucidated by administering the compound intraperitoneally to ICR mice on day 5 after subcutaneous tumor establishment (29). Compared with both the control and DMSO-treated groups, 2’,3’-cGAMP markedly inhibited tumor growth and reduced tumor volume (**Figures 7A, C**). After a 1-week treatment regimen, 2’,3’-cGAMP effectively curtailed LUAD progression (**Figures 7B, D**).

The immunomodulatory effects of 2’,3’-cGAMP, a known STING agonist, were investigated by analyzing macrophage populations within the TME of ICR mice. Administering 1 mg/kg 2’,3’-cGAMP notably shifted the M1-to-M2 macrophage ratio. At 0.5 mg/kg, 2’,3’-cGAMP induced a more pronounced increase in M1 macrophages, with a clear distinction observed between the macrophage subtypes (**Figures 7E–I**). Subsequent immunofluorescence analyses revealed that inducible nitric oxide synthase was upregulated, whereas PRKDC expression was markedly downregulated following the treatment (**Figures 7J–L**). Furthermore, 2’,3’-cGAMP promoted the M0-to-M1 phenotype differentiation in macrophages while inhibiting their conversion to the M2 phenotype (**Figures 7M, N**), which further facilitated the apoptosis in LUAD cells and suppressed tumor proliferation (**Figures 7O, P**) (30).

### 3.7 The effect of SB505124 and 2’,3’-cGAMP on apoptosis in LUAD cells

**Figure 8:**
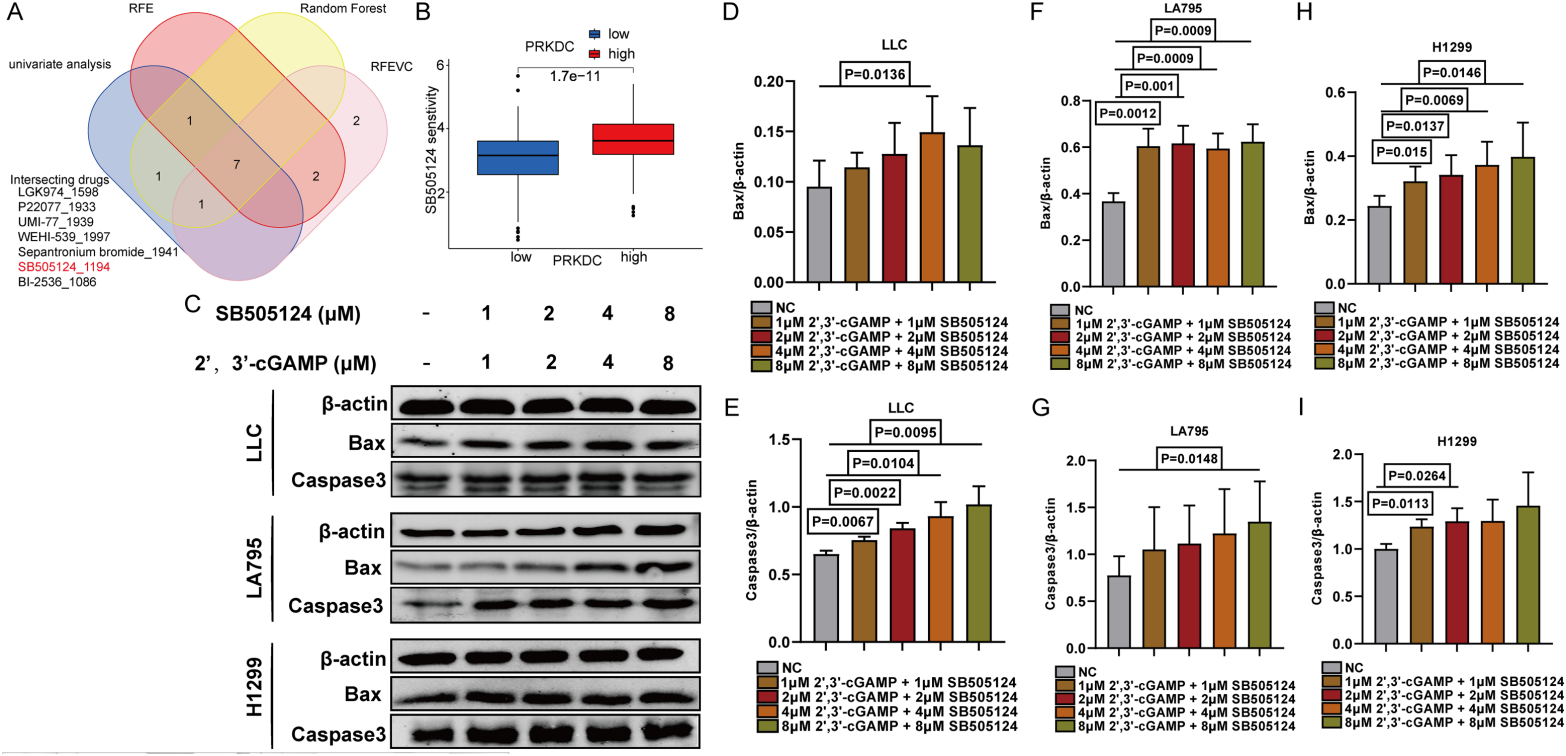
Screening of SB505124. **(A)** Venn diagram showed that seven drugs were correlated with PRKDC. **(B)** Association between susceptibility to SB505124 and PRKDC expression. **(C)** Western blot showed the apoptosis of LLC in co-culture of RAW264.7 and LUAD cells. **(D-I)** Apoptosis of co-cultured lung cancer cells.

Herein, seven PRKDC-associated pharmaceuticals were identified employing machine-learning approaches (**Figure 8A**). Among them, a targeted antagonist of transforming growth factor (TGF)-β1—a crucial secretory molecule produced by M2 macrophages—showed potential in inhibiting the M2 macrophage differentiation. SB505124 showed notable sensitivity in response to increased PRKDC expression (**Figure 8B**). Hence, lung cancer and macrophage cell lines were co-cultured to evaluate the combined effects of SB505124 and 2’,3’-cGAMP on apoptosis in LUAD cells. Western blotting revealed that SB505124 and 2’,3’-cGAMP synergistically increased the expression of apoptotic markers caspase-3 and Bax, thereby enhancing cell death in LUAD (**Figures 8C–I**).

### 3.8 The nano-delivery system efficiently exerted synergistic anti-LUAD activity via M1 macrophage polarization

**Figure 9:**
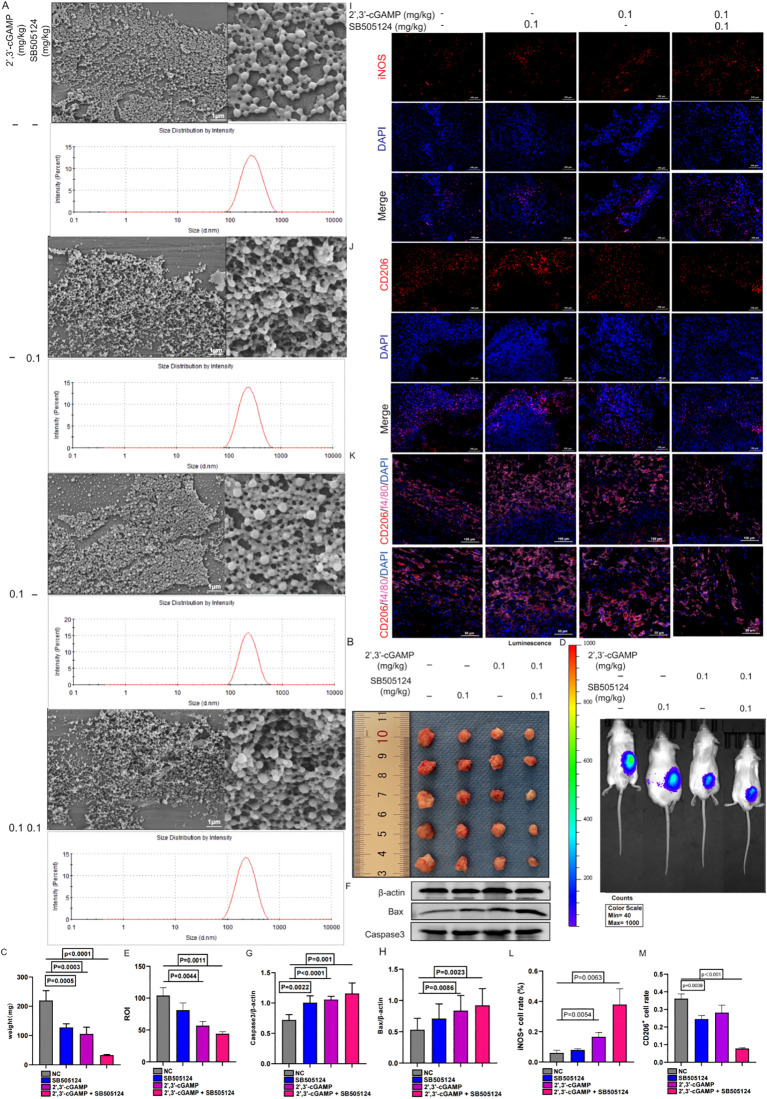
Characterization of nanosystems encapsulated in SB505124 and 2’,3’-cGAMP. **(A)** Sem images of typical nanoparticle, nanoparticle/SB505121, Nanoparticle/2’,3’-cGAMP, nanoparticle/SB505124 + 2’,3’-cGAMP, scale 1μm. **(B, C)** Local injection encapsulated SB505124 and 2’,3’-cGAMP (0.1mg/kg) nanospheres to inhibit tumor growth. **(D, E)** Representative bioluminescence image of local injection of nanospheres. **(F-H)** Tumor apoptosis of local injection of nanospheres. **(I-M)** Polarization of macrophages induced by comnination drugs.

After lyophilization, all PLGA nanoparticle formulations—whether loaded with SB505124, 2’,3’-cGAMP, or their combination or devoid of any drug—retained a consistent spherical morphology (**Figure 9A**). Localized administration of the SB505124 and 2’,3’-cGAMP nano-delivery system markedly inhibited tumor growth (**Figures 9B, C**), which was further verified by employing small animal imaging techniques (**Figures 9D, E**). Furthermore, the dual-drug treatment induced a more pronounced apoptotic response in tumors compared with that by single-drug treatments (**Figures 9F–H**). Immunofluorescence analyses revealed enhanced recruitment of M1 macrophages in the combination therapy, whereas the infiltration of M2 macrophages within LUAD tissues was reduced. Overall, these results indicate that SB505124 effectively inhibits TGF-β1, thereby reducing M2 macrophage polarization, and 2’,3’-cGAMP promotes M1 macrophage polarization, synergistically contributing to suppressing LUAD progression (**Figures 9I–M**).

## 4 Discussion

The findings of this study indicate PRKDC as a pivotal STING pathway-associated prognostic gene. PRKDC is involved in DNA repair and genomic stability, and in this study, PRKDC was upregulated in LUAD tumors and associated with poorer patient outcomes. Mendelian randomization analysis associated PRKDC expression with immune modulation within TME, highlighting its effect on apoptotic pathways and macrophage function (31, 32). The DNA-dependent protein kinase DNA repair complex has been reported to drive cyclic GMP–AMP synthase (cGAS)-independent interferon regulatory factor-3-mediated type I interferon response, and its catalytic activity is necessary for cGAS-dependent production of cGAMP and optimal downstream signal transduction (33).

Tumor-associated macrophages (TAMs) enhance cancer immunity by counteracting the immunosuppressive TME and specifically inhibiting antitumor T cell responses and reducing the effectiveness of immune checkpoint blockade therapies. In solid tumors, TAMs predominantly present an immunosuppressive phenotype; however, therapeutic interventions can trigger their inherent plasticity, allowing for phenotypic reprogramming and enabling the reversal of their suppressive state and subsequent hindering of tumor progression (34, 35). Herein, somatic mutation analysis revealed that the prevalence of TP53 and TTN mutations in the high-risk group was high, which is consistent with the findings reported in existing studies and associates these mutations with aggressive tumor phenotypes and poor prognosis in LUAD. Furthermore, the increased TMB observed in the high-risk group, particularly among patients with a high TMB and low risk, suggests a complex relationship between mutational load and immune surveillance. In the high-risk group, the mRNAsi was higher, indicating a more stem-like, aggressive tumor phenotype associated with therapeutic resistance and tumor recurrence. The results of immune profiling showed notable disparities in the expression of immune checkpoint proteins and HLA between the risk groups. In the low-risk group, immune-related pathways were enhanced, suggesting a more active antitumor immune response, whereas in the high-risk group, tumor-centric pathways were enhanced, potentially contributing to immune evasion. Overall, these findings emphasize the importance of immune contexture in LUAD and its potential as a therapeutic target to improve immunotherapy efficacy.

Reportedly, cGAS-mediated STING pathway activation initiates an immune response that enhances the efficacy of tumor-targeted immunotherapies (36) by upregulating programmed cell death ligand 1. Simultaneously, the cGAS–STING signaling cascade promotes the infiltration of T lymphocytes into M1-polarized macrophages, thereby emphasizing the intricate balance between immune activation and suppression within the TME (37). Similarly, in this study, the PRKDC–STING interaction was observed, which could be modulated by 2’,3’-cGAMP. Furthermore, 2’,3’-cGAMP facilitated the M0-to-M1 differentiation of macrophages while simultaneously inhibiting their transition to the M2 phenotype.

Functional assays further verified that the STING agonist 2’,3’-cGAMP effectively polarized macrophages toward the proinflammatory M1 phenotype, enhancing their cytotoxicity against LUAD cells and promoting apoptosis. Molecular docking results indicated that 2’,3’-cGAMP and SB505124 can interact with PRKDC binding sites, validating the results of machine-learning-based drug screening. The suppression is essential for the M2-to-proinflammatory state transformation of TAMs, which leads to the inhibition of tumor progression. Notably, macrophages aid in the restructuring and accumulation of the extracellular matrix within the TME by releasing inflammatory agents that trigger apoptosis in cancer cells (38). Herein, the combined treatment of SB505124, a TGF-β1 antagonist, with 2’,3’-cGAMP synergistically enhanced apoptotic marker expression and inhibited tumor growth, presenting a promising combinatorial therapeutic strategy.

Although accessible, effective cGAS agonists exhibit limited therapeutic potential owing to various reasons (33), which can be addressed by nanotechnology-based systems (39). The results of *in vivo* experiments revealed that using a dual-delivery platform containing SB505124 and 2’,3’-cGAMP encapsulated within PLGA nanoparticles markedly curtailed LUAD tumor growth compared with that of monotherapy approaches. Furthermore, immunofluorescence assays indicated that the combined treatment of SB505124 and 2’,3’-cGAMP successfully promoted the polarization of macrophages toward the M1 phenotype while decreasing the population of M2 macrophages. Consequently, this reprogrammed the TME to adapt to a more immunostimulatory state. Altogether, these findings indicate that SB505124 enhances the immunotherapeutic effectiveness of 2’,3’-cGAMP.

Future investigations need to focus on the role of PRKDC in other cancer types and its interaction with various components of the immune system to further elucidate the therapeutic scope of targeting PRKDC. Furthermore, the present limitations of PRKDC inhibitors, such as toxicity and solubility, need to be addressed through advancing drug delivery systems and rational drug design for translating these findings into effective clinical therapies. Nevertheless, this study has some limitations. Although PRKDC was identified as a key regulatory factor in LUAD progression and immune response, the mechanisms underlying the enhancement of the immune therapeutic effect of LUAD by reprogramming TAMs and the promotion of an antitumor immune environment remain elusive. The results of this study may serve as a basis to explore corresponding changes in mechanisms.

## 5 Conclusion

The findings of this study present a comprehensive prognostic model based on STING pathway-related genes and identify PRKDC as a key modulator of LUAD progression and immune response. SB505124 and 2’,3’-cGAMP exhibited notable synergistic therapeutic efficacy after being delivered by PLGA nanoparticles, offering a promising strategy to enhance immunotherapy outcomes in LUAD via the reprogramming of tumor-associated macrophages and promotion of an antitumor immune environment. Altogether, these findings may provide insights into the development of more effective and personalized treatment approaches for patients with LUAD.

## Data Availability

The original contributions presented in the study are included in the article/supplementary material. Further inquiries can be directed to the corresponding authors.
